# A new ELISA and western blot technique based on recombinant TES antigen and/or larval antigen for the detection of toxocariasis in humans

**DOI:** 10.1017/S0031182020002085

**Published:** 2021-03

**Authors:** Marie-Kristin Raulf, Daniela Jordan, Herbert Auer, Jens M. Warnecke, Bernd Lepenies, Christina Strube

**Affiliations:** 1 Institute for Parasitology, Centre for Infection Medicine, University of Veterinary Medicine Hannover, Buenteweg 17, 30559 Hannover, Germany; 2 Institute for Immunology, University of Veterinary Medicine Hannover, Buenteweg 17, 30559 Hannover, Germany; 3 Research Center for Emerging Infections and Zoonoses, University of Veterinary Medicine Hannover, Buenteweg 17, 30559 Hannover, Germany; 4 Department of Medical Parasitology, Center of Pathophysiology, Infectiology and Immunology, Institute of Specific Prophylaxis and Tropical Medicine, Medical University Vienna, Kinderspitalgasse 15, 1090 Vienna, Austria; 5 Institute for Experimental Immunology, EUROIMMUN, Seekamp 31, 23560 Lübeck, Germany

**Keywords:** Antibody detection, ELISA, epidemiology, immunoblot, serology, *Toxocara*, toxocariasis, toxocarosis, western blot

## Abstract

Serological antibody detection by enzyme-linked immunosorbent assay (ELISA)- and immunoblot-based methods constitutes the best indicator of human *Toxocara* infection. Nevertheless, the availability of serological tests, particularly western blots (WB), evaluated for sensitivity and specificity is limited. Therefore, an Anti-*Toxocara*-ELISA immunoglobulin g (IgG) prototype (Proto-ELISA) and an Anti-*Toxocara*-Westernblot (IgG) prototype (Proto-WB) were evaluated by testing 541 human sera pre-determined for *Toxocara* infection by an established *in-house* Anti-*Toxocara*-ELISA (IH-ELISA). To evaluate sensitivity and specificity of the newly developed ELISA and WB prototypes, results were compared to IH-ELISA and a commercial WB (Com-WB). Compared to the IH-ELISA, a sensitivity of 93.1% (229/246) and a specificity of 94.6% (279/295) of the Proto-ELISA with a Cohen's *κ* of 0.88 were obtained. The sensitivity of the Proto-WB was 76.7% (240/313) and specificity was 99.6% (227/228) with a Cohen's *κ* of 0.73 compared to those of Com-WB. A comparison to the IH-ELISA revealed 91.5% (225/246) sensitivity and 94.6% (279/295) specificity of the Proto-WB with a Cohen's *κ* of 0.86. Cross-reactivity was observed for some samples positive for *Ascaris* and *Trichinella* spp. in the Proto-ELISA, Proto-WB and Com-WB. Overall, the evaluated ELISA and WB prototypes showed high sensitivity and specificity, indicating high reliability of these newly developed tests.

## Introduction

The dog roundworm *Toxocara canis* and the cat roundworm *Toxocara cati* are worldwide-distributed zoonotic intestinal helminths, which infect humans as paratenic hosts. Persisting third-stage larvae (L3) may cause disease, including unspecific forms, the so-called covert toxocarosis, as well as visceral *larva migrans*, ocular *larva migrans* and neurotoxocarosis. In severe cases, especially in children under 5 years of age, blindness, eosinophilic meningitis, encephalitis or myelitis may occur (Strube *et al*., 2013). Due to a high global burden, the severity of the illness and the poor surveillance, prevention and treatment of the disease, toxocarosis constitutes a ‘neglected parasitic infection’ according to the Centers for Disease Control and Prevention (Woodhall *et al*., 2014; CDC, 2020). Reports of clinical cases are scarce; however, worldwide seroprevalence rates vary from 6.2% in Europe, 12.8% in the North Americas, 24.2% in the Western Pacific region, 27.8% in the South Americas, 34.1% in South-East Asia to 37.7% in Africa with an estimated global burden of 19.0% (Rostami *et al*., 2019; Ma *et al*., 2020; Strube *et al*., 2020). In recent years, seroprevalence rates tend to increase in Europe, possibly due to increasing dog and cat populations in combination with closer human–animal relationship and a change in recreational activities with more time spent in nature (Strube *et al*., 2020). Noteworthily, actual *Toxocara* exposure might be underestimated as a considerable amount of infected persons are asymptomatic (Noordin *et al*., 2020) and thus not tested. Furthermore, there are gaps in the epidemiology of *Toxocara* spp. infection as seroepidemiological data are still partly missing on global, national and regional scales, especially in low-income areas (Ma *et al*., 2020; Strube *et al*., 2020).

Most epidemiological data arise from enzyme-linked immunosorbent assay (ELISA) or immunoblot detection of anti-*Toxocara* antibodies in sera. Similarly, sera of patients suspected of toxocarosis are typically tested for individual diagnosis by ELISA, as a high throughput of samples with comparably low costs can be achieved with this method. Because western blotting (WB) often constitutes a highly specific detection method that tends to be less cross-reactive with pathogens other than *Toxocara* spp., it is thus frequently used to confirm ELISA-positive results (Smith and Noordin, 2006; Fillaux and Magnaval, 2013; Ma *et al*., 2020). However, sensitivity and specificity do not only depend on the applied method, but are also mainly affected by the utilized *Toxocara* antigens (embryonated egg-, larval-, excretory-secretory- or recombinant-antigens) and detected immunoglobulin classes (IgG and its subclasses, IgM, IgE, etc.) (Noordin *et al*., 2020). For instance, somatic antigen extracts of adult *T. canis* worms are highly cross-reactive, especially with *Ascaris* spp., whereas the use of larval excretory-secretory antigens improved ELISA specificity (de Savigny *et al*., 1979; Jacquier *et al*., 1991; Wilkins, 2014). In recent years, attention has been paid to the detection of specific IgG subclasses, with IgG2 being most sensitive and IgG3 and IgG4 being more specific compared to total IgG (Noordin *et al*., 2005; Smith and Noordin, 2006; Watthanakulpanich *et al*., 2008). Overall, different combinations of the abovementioned factors have resulted in the development of manifold ELISA variants since the late-1970s and WB variants since the late-1980s (de Savigny *et al*., 1979; Magnaval *et al*., 1991; Noordin *et al*., 2020). Hence, several ELISA and a few WB kits are commercially available with varying diagnostic sensitivities and specificities ranging from 80 to 100% (Smith and Noordin, 2006; Hamilton *et al*., 2014). Of these, many refer to determination of internal specificity and sensitivity, whereas published data in peer-reviewed journals are less common.

Recently, a new ELISA and WB have been developed [Anti-*Toxocara*-ELISA (IgG) prototype and Anti-*Toxocara*-Westernblot (IgG) prototype, EUROIMMUN]. Here, the performance of these assays was evaluated by testing human sera pre-determined for *Toxocara* seropositivity. Additionally, examined sera included samples from patients positive for parasitoses other than toxocarosis to test for potential cross-reactions of the newly developed serodiagnostic assays.

## Materials and methods

### Human sera and pre-determination

This study included 541 human serum samples that were tested at the Institute of Specific Prophylaxis and Tropical Medicine, Medical University Vienna, Austria, between 2014 and 2018 for diagnostic purposes. These samples were pre-determined by a *Toxocara* excretory-secretory (TES) antigen-based *in-house* Anti-*Toxocara*-ELISA (IH-ELISA), and positive ELISA results were confirmed by a TES antigen-based *in-house* Anti-*Toxocara*-WB (IH-WB) (Schneider *et al*., 2015) as part of the institute's routine diagnostic. Of these 541 sera, 246 were *Toxocara*-seropositive in both IH-ELISA and IH-WB, whereas 295 were seronegative in the IH-ELISA. The 295 negative sera included 45 samples that were seropositive for other parasites, i.e. *Ascaris* spp. (31 samples), *Trichinella* spp. (four samples), *Fasciola* spp. (five samples), *Schistosoma* spp. (two samples), *Echinococcus* spp. (two samples) and *Entamoeba* spp. (one sample), and were used to assess the potential cross-reactivity of the tests evaluated in this study. Furthermore, 11 additional samples seropositive for *Schistosoma* spp. (one sample), *Echinococcus* spp. (six samples), *Taenia* spp. (cysticercosis, one sample) and *Entamoeba* spp. (three samples) were available for cross-reactivity testing; however, these samples were not pre-determined for anti-*Toxocara* antibodies.

### Anti-*Toxocara*-ELISA (IgG) prototype (Proto-ELISA)

The Anti-*Toxocara*-ELISA (IgG) prototype (Proto-ELISA, EUROIMMUN, Lübeck, Germany, cat no. EI 2311-9601 G) was performed as recommended by the manufacturer. Briefly, microtitre plates coated with *T. canis* soluble larval somatic antigen (purified from egg-hatched L3) and recombinant 30 kDa TES antigen were incubated with sera in a dilution of 1:101 in sample buffer for 1 h at 37°C. For semi-quantitative analysis, a calibrator, a positive control and a negative control were included in each run. After incubation, plates were washed three times with washing buffer for 30–60 s at room temperature (RT). Afterwards, wells were incubated with rabbit anti-human IgG conjugated to horseradish-peroxidase for 30 min at 37°C followed by washing as described above. Colorimetric detection was initiated by applying tetramethylbenzidine substrate solution for 30 min at RT. The reaction was stopped by the addition of 0.5 m sulphuric acid and optical density (OD) was measured using a Biowave 340 photometer (BioTek, VT, USA) at a wavelength of 450 nm as well as at a reference wavelength of 620 nm to exclude background signals. A semi-quantitative signal-to-cut-off ratio of tested samples was calculated using the calibrator (extinction of sample/extinction of calibrator). Samples were tested negative, borderline or positive if the OD ratio was <0.8, ⩾0.8 to <1.1 or ⩾1.1, respectively.

### Anti-*Toxocara*-Westernblot (IgG) prototype (Proto-WB)

**Fig. 1.**
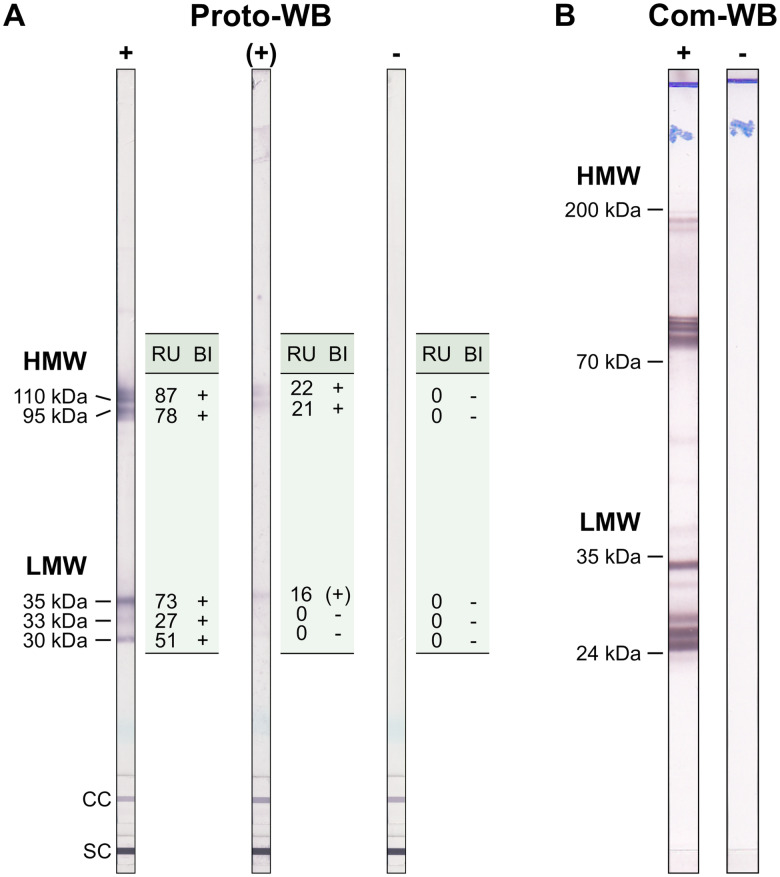
Example results of (A) the *Toxocara* Proto-WB and (B) the Com-WB. Bands of lower molecular weight are specific for anti-*Toxocara* antibodies. For Proto-WB, results of each of the bands were categorized (band interpretation, BI) based on RUs. +, positive; (+), borderline; −,negative; LMW, low-molecular weight; HMW, high-molecular weight; SC, serum control; CC, conjugate control.

The Anti-*Toxocara*-Westernblot (IgG) prototype (Proto-WB, EUROIMMUN) is based on electrophoretically separated larval (L3) somatic antigen of *T. canis*. A serum control membrane chip and an IgG conjugate control membrane chip were included in each WB strip. Furthermore, a validation strip (cat no. DL 0160-1601 G) was included in each run to ensure proper assay performance. The Proto-WB was performed as recommended by the manufacturer. Briefly, strips were blocked with universal buffer (cat no. ZW 1100-1005) for 15 min and then incubated with serum samples in a dilution of 1:51 in universal buffer for 30 min at RT. After three washing steps with universal buffer for 5 min, strips were incubated with polyclonal goat anti-human IgG conjugated to alkaline phosphatase (AP; cat no. AE 142-1030) for 30 min at RT followed by another three times washing step as described above. Antibody binding was visualized by incubation with nitro blue tetrazolium chloride/5-bromo-4-chloro-3-indolyl phosphate (NBT/BCIP; cat no. ZW 1020-0130) for 10 min and the enzyme reaction was stopped by washing three times with distilled water for 1 min. Strips were analysed with the EUROLineScan software (EUROIMMUN) using the flatbed scanner CanoScan LiDE 110 (Canon, Tokyo, Japan). Band intensity was converted to a relative unit (RU), with RUs ⩾19 considered positive. RUs ranging from ⩾12 to <19 indicated borderline results. Bands were classified into two groups: a triplet band at low-molecular weight (30, 33 and 35 kDa) being specific for anti-*Toxocara* antibodies, and two bands at high-molecular weight (95 and 110 kDa; Fig. 1A) detecting antibodies against *Toxocara* spp. and also other parasites. A sample was defined as positive if at least one of low-molecular weight bands showed a positive RU. Borderline results were characterized by one low-molecular weight band displaying a borderline RU and at least one of the high-molecular bands showing a positive RU (Fig. 1A).

### *Toxocara* Western Blot IgG (Com-WB)

To assess quality parameters of the Proto-WB, results were compared to those of *Toxocara* Western Blot IgG (Com-WB; LDBIO Diagnostics, Lyon, France, cat no. TOX-WB-24). The Com-WB is based on electrophoretically separated larval TES antigen of *T. canis* and intended for confirmatory testing of a positive or equivocal result obtained using classic screening tests. It was performed as described in the manufacturer's instructions. First, strips were rehydrated in sample buffer for 1 min followed by the addition of serum in a final dilution of 1:121 for 90 min at RT. A positive control serum was included in each run. Membranes were washed three times for 3 min with washing buffer and were incubated with AP-labelled anti-human IgG conjugate solution for 60 min at RT. After another three times washing step as described above, colorimetric detection was initiated by incubation with NBT/BCIP substrate solution and stopped after 60 min by washing two times with distilled water. Results were evaluated visually. The bands present at low-molecular weights (between 24 and 35 kDa) were specific for antibodies against *Toxocara* spp., whereas the bands at higher molecular weights (between 70 and 90 kDa and 100 and 200 kDa) indicated the presence of antibodies against *Toxocara* spp. or other parasites (Fig. 1B). According to the manufacturer, a simultaneous appearance of at least two bands in the low-molecular weight area defines *Toxocara* seropositivity. Results difficult to interpret, i.e. due to faint bands, were evaluated by two additional investigators to increase objectivity.

### Statistics

Sensitivity, specificity, the positive predictive value (PPV) and the negative predictive value (NPV) of the newly developed Proto-ELISA and Proto-WB were determined. This was achieved by comparing results of the Proto-ELISA against those of the IH-ELISA, and of the Proto-WB against the Com-WB. The abovementioned quality parameters for the Proto-ELISA and the Proto-WB were calculated as previously described (Trevethan, 2017). Furthermore, sensitivity, specificity, PPV and NPV were calculated by comparing results of the Proto-WB against those of the IH-ELISA. However, these results were not included in the assessment of quality parameters. Cohen's *κ* was calculated to determine inter-rater agreement of the two respective testing methods (Cohen, 1960). As borderline samples in both Proto-ELISA and Proto-WB are omitted in formulas calculating quality parameters and Cohen's *κ*, we considered these samples positive for reintegration. Goodness-of-fit of linear regression analysis of Proto-ELISA and IH-ELISA results was determined by computing *R*^2^. Furthermore, Spearman's rank correlation coefficient (*r*) provided information about the correlation of ELISA and WB results. The results of Proto-WB (1 = positive, 0.5 = borderline, 0 = negative) and Com-WB (1 = positive, 0 = negative) were encoded for correlation analysis. Specificity, sensitivity, PPV, NPV and Cohen's *κ* were computed by using Microsoft^®^ Excel^®^ (Version 2016, Redmond, Washington, USA), whereas GraphPad Prism™ (Version 8.0, La Jolla, California, USA) was used for statistical analysis of *R*^2^, Spearman's rank correlation and the respective coefficients.

## Results

### Sensitivity, specificity and cross-reactivity of the Proto-ELISA

According to the Proto-ELISA, 245 [with 223 (41.2%) samples being positive and 22 (4.1%) being borderline] of the 541 serum samples were positive and 296 (54.7%) negative for antibodies against *Toxocara* species. Results of the pre-determination were comparable, with 246 (45.5%) samples being positive and 295 (54.5%) being negative in IH-ELISA (Table 1). Sensitivity was calculated on the 246 sera pre-determined as *Toxocara*-positive. Of the 246 samples, 229 were positive in both Proto-ELISA and IH-ELISA, resulting in a Proto-ELISA sensitivity of 93.1% (229/246). The PPV, which is based on the 245 *Toxocara*-positive samples in Proto-ELISA, reached 93.5% (229/245), with 16 samples being false-positive in Proto-ELISA compared to IH-ELISA. Ten (62.5%) of these 16 false-positive samples were positive in all other testing procedures, i.e. Proto-ELISA, Proto-WB and Com-WB, four (25.0%) positive in both Proto-ELISA and Com-WB and two (12.5%) positive in the Proto-ELISA only.

**Table 1.** Sensitivity and specificity of the newly developed *Toxocara* Proto-ELISA and Proto-WB compared to pre-determination results of the 541 human sera by IH-ELISA and Com-WB

		Positive	Negative	Sensitivity (%)	Specificity (%)
IH-ELISA *vs*					
Proto-ELISA	Positive/borderline (total)	214/15 (229)	9/7 (16)	93.1	94.6
	Negative	17	279		
Com-WB *vs*					
Proto-WB	Positive/borderline (total)	228/12 (240)	1/0 (1)	76.7	99.6
	negative	73	227		
IH-ELISA *vs*					
Proto-WB	Positive/borderline (total)	217/8 (225)	12/4 (16)	91.5	94.6
	negative	21	279		

Specificity was calculated on the 295 sera pre-determined as negative. A total of 279 samples were negative in both Proto-ELISA and IH-ELISA, resulting in a Proto-ELISA specificity of 94.6% (279/295). The NPV, calculated on the 296 samples negative in Proto-ELISA, was 94.3% (279/296) with 17 sera that were negative in the Proto-ELISA despite being positive in IH-ELISA. Of these, two (11.8%) samples were negative in the Proto-ELISA, Proto-WB and Com-WB, 11 (64.7%) were negative in both Proto-ELISA and Proto-WB and four (23.5%) were negative in Proto-ELISA only.

**Fig. 2.**
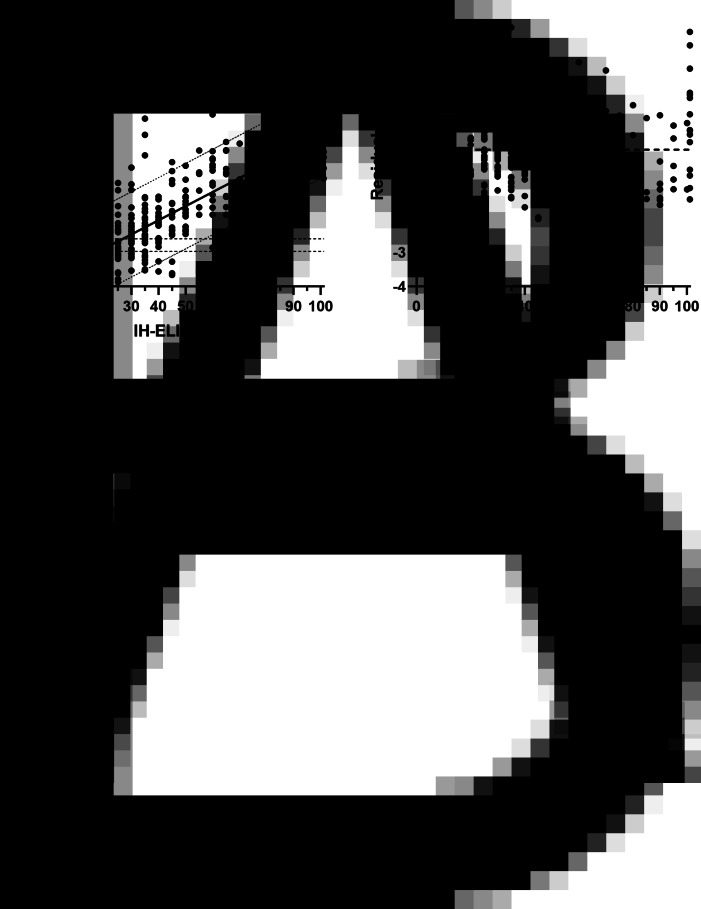
Correlation of the *Toxocara* reactivity of 541 human serum samples analysed in the Proto-ELISA and the IH-ELISA. (A) RUs of the Proto-ELISA were plotted against antibody units (AU) of the IH-ELISA with subsequent calculation of *R*^2^ as indicated by the line of best fit (thick line) and 95% prediction lines (dotted lines). Horizontal dashed lines indicate cut-off values for borderline (⩾0.8 to >1.1) and positive (⩾1.1) results of the Proto-ELISA. (B) Residuals of the regression line plotted against the AU of the IH-ELISA. Equal distribution of the residuals around the regression line (dashed line) indicates accuracy of the applied regression model.

Accordingly, the inter-rater agreement indicated a substantial accordance of Proto-ELISA and IH-ELISA with a Cohen's *κ* of 0.88. Semi-quantitative analysis of results is possible in Proto- and IH-ELISA due to calibrator samples and the calculation of arbitrary units as an indicator for *Toxocara* reactivity. Therefore, linear regression analysis was conducted by computing *R*^2^, the coefficient of determination to a regression line of *Y* = 0.04641*X* + 0.3217, with a value of 0.79 (*P* < 0.0001) (Fig. 2A). Residuals were equally distributed around the regression line as an indicator for the accuracy of the model (Fig. 2B). Furthermore, Spearman's rank correlation exhibited a value of *r* = 0.86 (*P* < 0.0001). Thus, results indicate a high goodness-of-fit and a high correlation of both testing methods.

**Fig. 3.**
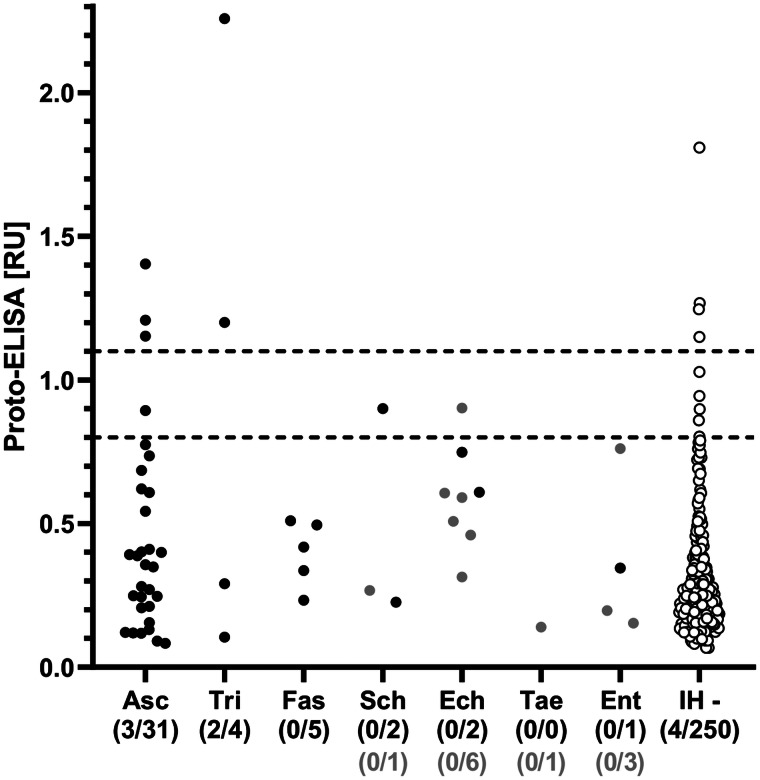
Reactivity of the *Toxocara* Proto-ELISA to 56 serum samples positive for parasites other than *Toxocara* spp. (black dots: 45 *Toxocara*-negative sera by the IH-ELISA; grey dots: 11 sera not pre-determined for *Toxocara* seropositivity by IH-ELISA) and 250 sera tested negative by the IH-ELISA (white dots, IH -). Horizontal dashed lines indicate cut-off values for borderline (⩾0.8 to >1.1) and positive (⩾1.1) results of the Proto-ELISA. Asc, *Ascaris* spp.; Tri, *Trichinella* spp.; Fas, *Fasciola* spp.; Sch, *Schistosoma* spp.; Ech, *Echinococcus* spp.; Tae, *Taenia* spp. (cysticercosis); Ent, *Entamoeba* species.

Cross-reactivity was evaluated based on 45 samples that were seropositive for *Ascaris*, *Trichinella*, *Fasciola*, *Schistosoma*, *Echinococcus* and *Entamoeba* spp., but negative for *Toxocara* spp. in the IH-ELISA. Overall, cross-reactivity in the Proto-ELISA was observed for 11.1% (5/45) of samples. Of these, 3/31 (9.7%) patients that were seropositive for *Ascaris* spp. and 2/4 (50.0%) patients seropositive for *Trichinella* spp. showed cross-reactivity. Borderline results were observed in 4.4% (2/45) of samples, namely in 1/31 (3.2%) patients suffering from *Ascaris* and 1/2 (50.0%) from *Schistosoma* spp. infections (Table 2). Samples positive for antibodies against *Fasciola* and *Entamoeba* spp. showed no cross-reactivity. Of the additional 11 samples not pre-determined by IH-ELISA, only one patient serum positive for *Echinococcus* spp. displayed borderline results in the Proto-ELISA. Proto-ELISA-derived RUs of the potentially cross-reactive sera as well as the pre-determined negative sera are depicted in Fig. 3. Overall, results indicate a high sensitivity and specificity with more than 90.0% and a low cross-reactivity of the newly developed Proto-ELISA.

**Table 2.** Cross-reactivity of the *Toxocara* Proto-ELISA with 45 samples positive for antibodies against parasites other than *Toxocara* species

	Proto-ELISA		
	Positive [% (95% CI)]	Borderline [% (95% CI)]	Negative [% (95% CI)]
*Ascaris*	3/31 [9.7 (3.3–24.9)]	1/31 [3.2 (0.2–16.2)]	27/31 [87.1 (71.1–94.9)]
*Trichinella*	2/4 [50.0 (8.9–91.1)]	0/4 [0.0 (0.0–49.0)]	2/4 [50.0 (8.9–91.1)]
*Fasciola*	0/5 [0.0 (0.0–43.4)]	0/5 [0.0 (0.0–43.4)]	5/5 [100.0 (56.6–100)]
*Schistosoma*	0/2 [0.0 (0.0–82.2)]	1/2 [50.0 (2.6–97.4)]	1/2 [50.0 (2.6–97.4)]
*Echinococcus*	0/2 [0.0 (0.0–82.2)]	0/2 [0.0 (0.0–82.2)]	2/2 [100 (17.8–100)]
*Entamoeba*	0/1 [0.0 (0.0–94.9)]	0/1 [0.0 (0.0–94.9)]	1/1 [100 (5.1–100)]
Total	5/45 [11.1 (4.8–23.5)]	2/45 [4.4 (0.8–14.8)]	38/45 [84.4 (71.2–92.3)]

CI, confidence interval.

### Sensitivity, specificity and cross-reactivity of the Proto-WB

In the Proto-WB, 241 [composed of 229 positive (42.3%) and 12 (2.2%) borderline samples] of the 541 serum samples were positive and 300 (55.5%) negative for anti-*Toxocara* antibodies. Sensitivity, specificity, PPV and NPV of the Proto-WB were calculated based on Com-WB as the reference. In the Com-WB, 313 (57.9%) samples were positive and 228 (42.1%) negative for antibodies against *Toxocara* species. Out of the 313 samples positive in Com-WB, 228 sera were positive in the Proto-WB as well. Sensitivity of the Proto-WB was rather low with 76.7% (240/313), whereas the PPV reached 99.6% (240/241) with only one false-positive sample. A total of 227 sera were negative in both Proto-WB and Com-WB, leading to a specificity of 99.6% (227/228), whereas the NPV was only 75.7% (227/300) due to 73 samples being false-negative in the Proto-WB (Table 1). A Cohen's *κ* of 0.73 indicated a substantial accordance, and Spearman's rank correlation with *r* = 0.75 (*P* < 0.0001) showed a significant correlation of both testing methods.

Besides the comparison to the Com-WB as the reference, sensitivity was additionally calculated based on the 246 human sera positive in IH-ELISA. Of the 246 samples, 225 sera were positive in Proto-WB and IH-ELISA (Table 1), resulting in a sensitivity of 91.5% (225/246). The PPV was 93.4% (225/241), with 16 samples being false-positive in the Proto-WB. However, 10 (62.5%) of these 16 samples were positive in Proto-ELISA, Proto-WB and Com-WB, and six (37.5%) sera were positive in both Proto-WB and Com-WB.

Specificity was calculated on the 295 sera pre-determined as negative by IH-ELISA. A specificity of 94.6% (279/295) resulted from 279 sera being negative in both Proto-WB and IH-ELISA. The NPV was 93.0% (279/300). Hence, 21 sera depicted a contrary negative result compared to IH-ELISA, of which two (9.5%) samples were negative in the Proto-WB, Proto-ELISA and Com-WB, 11 (52.4%) negative in the Proto-WB and Proto-ELISA and eight (38.1%) negative in the Proto-WB only. The inter-rater agreement indicated a substantial accordance of the Proto-WB and IH-ELISA with a Cohen's *κ* of 0.86.

Determination of cross-reactivity based on 45 pre-determined samples that were seropositive for parasites other than *Toxocara* identified only 6.7% (3/45) as cross-reactive in the Proto-WB. These were 2/31 (6.5%) samples from patients suffering from ascarosis and 1/4 (25.0%) patient suffering from trichinellosis. Additionally, borderline results were observed for 1/31 (3.2%) *Ascaris* spp.-positive sera, whereas no cross-reactivity was observed for *Fasciola*, *Schistosoma*, *Echinococcus* and *Entamoeba* spp. infections (Table 3). None of the 11 samples not pre-determined for *Toxocara* seropositivity was positive or borderline in the Proto-WB.

**Table 3.** Cross-reactivity of the *Toxocara* Proto-WB and Com-WB with 45 samples positive for antibodies against parasites other than *Toxocara* species

	Proto-WB			Com-WB	
	Positive [% (95% CI)]	Borderline [% (95% CI)]	Negative [% (95% CI)]	Positive [% (95% CI)]	Negative [% (95% CI)]
*Ascaris*	2/31 [6.5 (1.1–20.7)]	1/31 [3.2 (0.2–16.2)]	28/31 [90.3 (75.1–96.7)]	11/31 [35.5 (21.1–53.1)]	20/31 [64.5 (46.9–78.9)]
*Trichinella*	1/4 [25.0 (1.3–69.9)]	0/4 [0.0 (0.0–49.0)]	3/4 [75 (30.1–98.7)]	2/4 [50.0 (8.9–91.1)]	2/4 [50.0 (8.9–91.1)]
*Fasciola*	0/5 [0.0 (0.0–43.4)]	0/5 [0.0 (0.0–43.4)]	5/5 [100 (56.6–100)]	1/5 [20.0 (1.0–62.4)]	4/5 [80.0 (37.6–99.0)]
*Schistosoma*	0/2 [0.0 (0.0–82.2)]	0/2 [0.0 (0.0–82.2)]	2/2 [100 (17.8–100)]	1/2 [50.0 (2.6–97.4)]	1/2 [50.0 (2.6–97.4)]
*Echinococcus*	0/2 [0.0 (0.0–82.2)]	0/2 [0.0 (0.0–82.2)]	2/2 [100 (17.8–100)]	2/2 [100.0 (17.8–100.0)]	0/2 [0.0 (0.0–82.2)]
*Entamoeba*	0/1 [0.0 (0.0–94.9)]	0/1 [0.0 (0.0–94.9)]	1/1 [100 (5.1–100)]	1/1 [100 (5.1–100)]	0/1 [0.0 (0.0–94.9)]
Total	3/45 [6.7 (2.3–17.9)]	1/45 [2.2 (0.1–11.6)]	41/45 [91.1 (79.3–96.5)]	18/45 [40.0 (27.0–54.5)]	27/45 [60.0 (45.5–73.0)]

CI, confidence interval.

Cross-reactivity was also investigated for the Com-WB based on the IH-ELISA as the reference. In total, 40.0% (18/45) of the pre-determined samples were cross-reactive in the Com-WB, including all parasitoses. More specifically, 11/31 (35.5%) *Ascaris*-, 2/4 (50.0%) *Trichinella*-, 1/5 (20.0%) *Fasciola*-, 1/2 (50.0%) *Schistosoma*-, 2/2 (100%) *Echinococcus*- and 1/1 (100%) *Entamoeba* spp.-positive sera exhibited positive *Toxocara* reactivity in the Com-WB. Detailed results are shown in Table 3. Furthermore, two of the 11 not pre-determined samples, i.e. one *Echinococcus*- and one *Entamoeba* spp.-positive serum reacted positive in the Com-WB.

## Discussion

Until today, serological detection of antibodies is the best approach to assess *Toxocara* exposure in epidemiological studies. Although seropositivity does not necessarily reflect an acute infection, seroconversion in concordance with clinical and haematological signs renders a current infection highly probable in diseased patients (Van Den Broucke *et al*., 2015). Nevertheless, histopathological examination with morphological identification of larvae or the detection of parasite DNA in patient samples constitutes the only ways to prove an acute infection with certainty (Smith and Noordin, 2006). However, these elaborate methods are often not sufficiently sensitive and more invasive than antibody detection in blood serum, thus serological status in concordance with clinical data represents the best suitable indicators of *Toxocara* infection in humans (Pawlowski, 2001; Rubinsky-Elefant *et al*., 2010; Ma *et al*., 2020; Strube *et al*., 2020). Several commercial serological tests are available. However, procedures or results of determining the sensitivity and specificity of these tests are not commonly published. Here, we evaluated an Anti-*Toxocara*-IgG ELISA (Proto-ELISA) based on a mixture of larval somatic- and recombinant larval TES-antigen as well as an Anti-*Toxocara*-IgG WB (Proto-WB) based on larval somatic antigen, which have recently been brought to market. These assays were compared to an *in-house* ELISA (IH-ELISA) which has been in use for diagnostic purposes at the Institute of Specific Prophylaxis and Tropical Medicine of the Medical University Vienna, Austria, for more than two decades (Schneider *et al*., 2015) as well as to an established commercial WB (Com-WB) (Logar *et al*., 2004; Nicoletti *et al*., 2008; Qualizza *et al*., 2011; Zibaei *et al*., 2013; Artinyan *et al*., 2014; Despreaux *et al*., 2016; Lötsch *et al*., 2016).

Testing of the pre-determined samples by Proto-ELISA indicated a high sensitivity of 93.1% and specificity of 94.6%, which is in accordance with the values obtained for ELISAs established in other studies and/or developed by other manufacturers (Smith and Noordin, 2006; Noordin *et al*., 2020). Jin *et al*. (2013) designed an ELISA based on larval somatic antigen displaying a sensitivity of 92.2% and specificity of 86.6%. ELISAs that are based on recombinant larval *Toxocara* antigens show varying sensitivities and specificities ranging from 80.0 to 93.3% and 89.6 to 96.2%, respectively (Norhaida *et al*., 2008; Mohamad *et al*., 2009). To the best of our knowledge, the Proto-ELISA is the only serological method that uses both larval somatic- and recombinant larval TES-antigen for the detection of anti-*Toxocara* antibodies. Thus, no direct comparative values exist. The choice of diagnostic antigens, e.g. embryonated egg-, adult as well as larval somatic- and larval TES-antigen, has been the subject of discussion ever since long-time cultivation of *Toxocara* larvae was established, making high amounts of TES antigen available (de Savigny, 1975). To date, serological detection methods are mainly based on larval TES antigens due to improved sensitivity, specificity and low cross-reactivity with antibodies against parasitic infections other than *Toxocara*. Notably, the Proto-ELISA utilizing larval somatic antigen showed only slight cross-reactivity to *Ascaris*- and *Trichinella*-positive sera, even though results should be treated with some caution due to rather low sample sizes of some potentially cross-reactive parasite infections. Cross-reactions of nematode-directed antibodies to *Toxocara* antigens are commonly reported in the literature (Lynch *et al*., 1988; Jacquier *et al*., 1991). The observed low cross-reactivity in the Proto-ELISA might be attributed to the supplementation of recombinant larval TES antigens, which are known to reduce cross-reactions and therefore elevate the specificity of serological assays (Yamasaki *et al*., 2000; Wickramasinghe *et al*., 2008; Mohamad *et al*., 2009; Yunus *et al*., 2018). For instance, native TES-120 is cross-reactive, whereas in *Escherichia coli* or *Pichia pastoris* recombinantly expressed protein obtains a high diagnostic specificity, possibly due to altered glycosylation (Fong *et al*., 2003; Fong and Lau, 2004; Mohamad *et al*., 2009; Wilkins, 2014). Furthermore, recombinant expression of proteins allows the production of highly purified and standardized antigens, certainly contributing to the reproducibility and reliability of serological assays (Smith and Noordin, 2006; Wilkins, 2014; Noordin *et al*., 2020). Another factor supporting the reliability of the Proto-ELISA is the correlation of RUs with those of the reference ELISA. Overall, the obtained results indicate that the Proto-ELISA is a promising alternative for native TES-based ELISAs due to comparable sensitivity and specificity.

Similar to the Proto-ELISA and its IH-ELISA reference, antigenic sources of the Proto-WB and the reference Com-WB differed in terms of employing larval somatic antigen and larval TES antigen, respectively. Interestingly, both tests exhibit banding patterns with various bands present at a low- and high-molecular range, with specific reactivity to *Toxocara* spp. in the low-molecular range, whereas bands of higher molecular weight are known to be unspecific (Jin *et al*., 2013; Wilkins, 2014). The possibility of discrimination between specific and unspecific reactions contributes to the frequently reported elevated specificity of WBs compared to ELISAs. Thus, WB is often used as a confirmatory test of ELISA-positive results (Smith and Noordin, 2006; Fillaux and Magnaval, 2013; Ma *et al*., 2020). The calculated Proto-WB specificity of 99.6% is comparable, considering the underlying sample size, to the 100% reported by the manufacturer for the Com-WB serving as the reference for the quality parameter calculations. Nevertheless, the Proto-WB constitutes a suitable confirmatory assay.

Overall, specificity is strongly affected by cross-reactions to antibodies against other parasites. When testing the potentially cross-reactive sera pre-determined to be *Toxocara*-negative but positive for other parasitoses by IH-ELISA, cross-reactivity in the Proto-WB was observed in 6.7% of samples, whereas approximately six times more samples (40.0%) cross-reacted in the Com-WB. However, it cannot be ruled out that these cross-reactive samples might indeed be positive for *Toxocara* antibodies. According to Jacquier *et al*. (1991) there are problems to exclude the presence of *Toxocara* antibodies in sera used for the determination of cross-reactivity, hence they might have been unrecognized by the IH-ELISA. Such polyparasitism is commonly reported in tropical regions, in which the simultaneous infection with various helminths, especially those that are soil-transmitted, represents a tremendous challenge for *Toxocara* serology due to cross-reactions (Smith and Noordin, 2006; Fillaux and Magnaval, 2013; Wilkins, 2014; Ma *et al*., 2020; Noordin *et al*., 2020). Nevertheless, *Toxocara* seronegativity in most of the IH-ELISA pre-determined potentially cross-reactive samples was also shown by the Proto-ELISA and Proto-WB. Thus, it cannot be excluded that at least some of the 40.0% cross-reactivities in the Com-WB are false positives. This could be due to an excessive substrate incubation period during the Com-WB procedure (1 h compared to 5–10 min in other WBs), possibly provoking unspecific reactions. Also, interpretation of results of the Com-WB may be challenging due to the visual band assessment, which is subjective and/or depends on the visual capacity in case of faint bands. In contrast, the Proto-WB is analysed by using corresponding software excluding bands below a certain intensity threshold, leading to increased objectivity as results are not dependent on subjective impressions or visual capacity of the examiner. Overall, further evaluations are needed to clarify the contradictory cross-reaction results observed in the presented study.

With a calculated sensitivity of 76.7%, the Proto-WB seems to be less sensitive than the Com-WB. An *in-house* larval TES-based WB by Magnaval *et al*. (1991) is considered to have a 55% higher diagnostic sensitivity compared to designated commercial larval TES-ELISA kits (Gueglio *et al*., 1994; Courtade *et al*., 1995; Fillaux and Magnaval, 2013; Noordin *et al*., 2020). Thus, an elevated number of *Toxocara*-positive sera detected by the reference Com-WB, for which a comparable performance to the WB by Magnaval *et al*. (1991) is stated in the user manual, is explainable. However, as discussed above, the Com-WB detected a number of potentially cross-reactive sera to be *Toxocara*-positive. If these would be false positives, they would have negatively affected Proto-WB sensitivity. Hence, calculated sensitivity of the Proto-WB should be interpreted with some caution as the sensitivity of the reference Com-WB is not published, neither in the user manual nor in studies utilizing this test, and upon request, the manufacturer provided the information that sensitivity could not be computed due to the absence of a reference method. In general, comparison of quality parameters of different assays is challenging because the availability of sera derived from patients with direct and reliable detection of *Toxocara* larvae is very limited, whereas serologically pre-determined samples are easily accessible. As the sensitivity of the reference Com-WB is unknown and to gain a holistic overview, the Proto-WB was additionally compared to the pre-determination by IH-ELISA. Here, the calculated sensitivity of 91.5% and specificity of 94.6% were comparable to those of Proto-ELISA. Most of the samples that were either false-positive or false-negative as compared to the IH-ELISA showed similar results in the Proto-WB and Proto-ELISA. This is to be expected as both assays utilize the same larval somatic antigen.

In conclusion, the newly developed Proto-ELISA and Proto-WB display comparable sensitivity and specificity to other serological tests for *Toxocara* infection available on the market or employed as *in-house* tests by research facilities, reference centres and others. Advantages of the Proto-ELISA are the use of standardized and highly purified recombinant TES antigens contributing to the reproducibility of the ELISA. Furthermore, the Proto-WB with its corresponding software circumvents potential examiner-dependent inconsistency in visual assessment of bands and thus allows a standardized and objective analysis of results. High sensitivity of the Proto-ELISA and high specificity of the Proto-WB render both tests applicable for practical application in routine diagnosis or seroepidemiological studies on toxocarosis. However, when utilized for routine diagnosis, it should be kept in mind that the presence of antibodies against *Toxocara* spp. alone does not indicate a current infection, but a combination of clinical, haematological and serological methods is required.
